# JColorGrid: software for the visualization of biological measurements

**DOI:** 10.1186/1471-2105-7-225

**Published:** 2006-04-27

**Authors:** Marcin P Joachimiak, Jennifer L Weisman, Barnaby CH May

**Affiliations:** 1 Department of Plant and Microbial Biology, 461 Koshland Hall, University of California Berkeley, CA 94720-3102, USA; 2 Department of Cellular and Molecular Pharmacology, University of California San Francisco, QB3 Room 403, 1700 4^th ^Street, San Francisco, CA 94158-2542, USA; 3 Institute for Neurodegenerative Diseases and Department of Neurology, University of California San Francisco, San Francisco, CA 94143-0518, USA

## Abstract

**Background:**

Two-dimensional data colourings are an effective medium by which to represent three-dimensional data in two dimensions. Such "color-grid" representations have found increasing use in the biological sciences (e.g. microarray 'heat maps' and bioactivity data) as they are particularly suited to complex data sets and offer an alternative to the graphical representations included in traditional statistical software packages. The effectiveness of color-grids lies in their graphical design, which introduces a standard for customizable data representation. Currently, software applications capable of generating limited color-grid representations can be found only in advanced statistical packages or custom programs (e.g. micro-array analysis tools), often associated with steep learning curves and requiring expert knowledge.

**Results:**

Here we describe JColorGrid, a Java library and platform independent application that renders color-grid graphics from data. The software can be used as a Java library, as a command-line application, and as a color-grid parameter interface and graphical viewer application. Data, titles, and data labels are input as tab-delimited text files or Microsoft Excel spreadsheets and the color-grid settings are specified through the graphical interface or a text configuration file. JColorGrid allows both user graphical data exploration as well as a means of automatically rendering color-grids from data as part of research pipelines.

**Conclusion:**

The program has been tested on Windows, Mac, and Linux operating systems, and the binary executables and source files are available for download at .

## Background

Scientific research requires data visualization for exploration and discovery, as well as in written and oral presentations. Biological science traditionally relies on manual analysis of curated datasets – an approach that is becoming increasingly challenging, as data sets and the volume of published material grow exponentially. Custom software combined with manual intervention have become necessary in order to efficiently manage these data sets, while providing the necessary results of analysis. Many methods of data management and analysis have been presented, however the techniques for data representation have remained largely unchanged. As a result, many custom and private solutions have been developed to satisfy data visualization needs.

**Figure 1:**
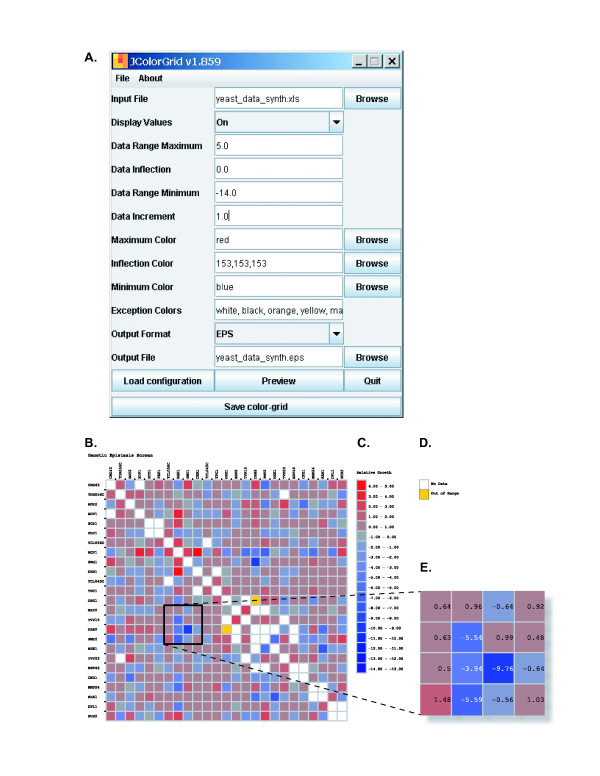
**JColorGrid parameters and graphical output**. **A**. The JColorGrid graphical configuration menu or text configuration files (not shown) allow users to create custom color-grids. The configuration shown was used to generate the color-grid shown in **B**., where synthetic lethal genetic interactions are shown in blue, suppressor interactions in red, and non-interactions in light gray. The color-grid output includes a color scale (**C**.) and exceptions key (**D**.). JColorGrid can be configured to either hide data values (**B**.), or display data values (**E**.) in the color-grid cells.

In our own work in the fields of computational biology and medicinal chemistry, color-grid representations have become increasingly useful to illustrate trends in data, whether they be correlations between features of pre-mRNA splice sites or structure-activity relationships (SAR) from large small-molecule libraries. Color-grids are two-dimensional data arrays where data values are represented visually by associated color intensities (see Figure 1). We were inspired in part by heat maps that effectively use color as an additional dimension, a technique successfully applied to microarray data visualization. However, the pioneering microarray data analysis software Cluster and TreeView [1] and recent extensions of this work [2-4] have some disadvantages when generalizing to a wider variety of datasets: a need for computational data manipulation, a focus on data clustering, incompatibility with text values and data exceptions, and limited coloring capabilities. We remedy some of these shortcomings with the design of an abstract color-grid graphical object and implement creation and rendering of such graphics in a program called, JColorGrid. The software is applicable to a wide variety of input data while retaining ease of use.

Data representation with a color-grid allows researchers and audiences to rapidly identify trends within large data sets. Color-grids follow the main graphical presentation tenets set forth by Edward Tufte: utilizing color to enhance information, facilitating micro and macro readings of data, graphical layering and separation, and use of "small multiple designs" for "graphical depictions of variable information that share context, but not content" [5]. Color-grids are data-dense and easily interpretable at different scales of analysis, making color-grids increasingly popular in the scientific literature. While color-grid representations are available in some advanced statistical packages (e.g. MatLab and R [6]), the commonly used free and commercial spreadsheet and statistical packages (e.g. StarOffice, MS Excel, SigmaPlot, KaleidaGraph, GnuPlot), do not offer color-grids as a graphical representation. It should be noted, that where available, color-grid outputs have limited utility due to the level of expertise necessary to work with these complex statistical packages. The absence of stand-alone software capable of automatically generating color-grids prompted us to develop JColorGrid, a Java application that serves as an engine for generating custom color-grid representations. Our motivation was to offer a novel, automated, general-purpose means to graphically represent complex data sets from various research disciplines following graphical visualization guidelines.

## Implementation

JColorGrid is a platform-independent pure Java application requiring Java 1.4 or higher. The software has been tested to function equally on MacOS X, Windows XP, and Linux RedHat 8 environments. The input data (values, color-grid title, scale title, column and row titles) are read by JColorGrid as a spreadsheet (i.e. .xls), or a tab-delimited text file. The program uses the JExcel API [7] to interface with MS Excel format spreadsheets. Data is formatted in the input file following a simple template that reflects the final color-grid layout (e.g Table 1). Color-grid color options can be specified with primary color text labels or 24 bit color using the Java Swing Color Picker or comma delimited RGB values.

JColorGrid has the ability to output JPEG or EPS format graphics, using functionality from the standard Java distribution and the Java EPS Graphics2D API [8], respectively. The EPS vector graphics format enables further color-grid graphic manipulation using vector graphics editing software. An input file with 40,000 data points requires approximately 30s of CPU time on a Pentium IV 1.7 GHz processor.

## Results

The spreadsheet and tab-delimited text formats were chosen for JColorGrid, as many scientists already manipulate data using these formats and thus have ready access to suitable software environments. The application can be configured and run either from the command line or through the graphical interface (Figure 1A). The command-line instantiation can be used to programmatically generate color-grids of multiple data sets with custom parameters. The graphical interface includes a color-grid preview window useful for graphical data exploration and visual assessment of the color-grid parameters. Color-grid options include: minimum, maximum, and inflection colors, scale increments, exceptions, and the format of graphical output (i.e. JPEG or EPS). Numerical data used to generate the color-grid can either be displayed in the output color-grid cells (e.g. Figure 1B), or hidden (e.g. Figure 1E), by using the 'Display Values' configuration (Figure 1A). By default JColorGrid will identify the minimum and maximum data points within a data set, and will map these data points to the extremes of the user defined color scale (Figure 1C). Users can override the default settings and specify a user defined data subrange. In the instance where data falls outside of the user defined range, JColorGrid will flag these outlying data points as 'Out of Range' and will color these data points to distinguish them from the data color scale (Figure 1D). This feature allows users to quickly identify data points that fall outside a specified range using color cues.

JColorGrid possesses separate and non-overlapping color mappings for text and numerical data, and therefore is not limited to purely numerical input. For instance, in the event JColorGrid reads an Excel file containing cells formatted as text, these text cells will be colored based on a set of configurable exception colors, distinct from the colors used in the color scale corresponding to the numerical data. JColorGrid treats any data value with non-numeric characters (excluding 'E','e' to denote exponents) as case-sensitive text and maps these values to the available exception colors. JColorGrid is limited to nine or ten unique text items in any data set, depending on the number of colors used in the numerical data color scale. The ability to process text data allows users to flag certain data points that require special color treatment within a larger numerical data set, a feature we term 'exceptions'. If they are present in a data set, these text exceptions are listed, along with the associated color, in an exceptions key (see 'No Data', Figure 1D). JColorGrid will generate color-grids containing either two or three colors mapped to the data range, as the color scale can be customized by specifying the inflection point for color transitions.

## Conclusion

Effective data presentation and analysis is an important facet of successful scientific research. The struggle to efficiently and accurately interpret data sets rapidly increasing in size requires consistent implementation of graphical standards in the form of accessible and robust tools. The color cues provided by color-grids aid in data analysis and serve as a platform for standardized data representation. JColorGrid is implemented as a convenient data visualization tool for generation of color-grids. The software helps overcome the limitations of manual graphics preparation and allows users to circumvent advanced or proprietary commercial tools by enabling customizable and automated data visualization.

## Availability and requirements

**Project name**: JColorGrid.

**Operating system**: Platform independent.

**Programming language**: Java.

**Other requirements**: None. A spreadsheet application (e.g. Excel) and/or a graphics editing software are optional but recommended.

**License**: Source code and a binary executable are available under terms of the GPL free software license (version 2 or later) at . Incorporation into commercial software under non-GPL terms is possible by obtaining a custom license from the University of California.

**URL**: 

## Authors' contributions

MPJ wrote the Java code. MPJ, JLW and BCHM conceived of the software, and contributed to its design. All authors contributed to the preparation of the manuscript, and have read and approved the final manuscript.

## Figures and Tables

**Table 1:** Example of biological input data suitable for color-grid representations. An example of the JColorGrid input data format. The format specifies numeric data points and text exceptions as well as optional elements such as the color-grid title, color-scale title, and column and row headings. Data shown in Table 1 is a subset (as indicated on the top left hand corner of the color-grid, Figure 1), of an epistasis genetic screen in yeast, where negative (synthetic lethal) and positive (suppressor) genetic interactions were measured [9]. A more complete dataset was used to generate the color-grid shown in Figure 1

	CWH43	YDR056C	ROT2	ADP1	SCT1	PEP1
CWH43	No Data	1.43	-1.29	No Data	0.08	1.31
YDR056C	1.43	No Data	1.39	1.23	0.81	0.23
ROT2	-1.29	1.39	No Data	0.74	0.31	-0.87
ADP1	No Data	1.23	0.74	No Data	0.25	-0.64
SCT1	0.08	0.81	0.31	0.25	No Data	No Data
PEP1	1.31	0.23	-0.87	-0.64	No Data	No Data

## References

[B1] Eisen MB, Spellman PT, Brown PO, Botstein D (1998). Cluster analysis and display of genome-wide expression patterns. Proc Natl Acad Sci U S A.

[B2] Saldanha AJ (2004). Java Treeview – extensible visualization of microarray data. Bioinformatics.

[B3] Saeed AI, Sharov V, White J, Li J, Liang W, Bhagabati N, Braisted J, Klapa M, Currier T, Thiagarajan M, Sturn A, Snuffin M, Rezantsev A, Popov D, Ryltsov A, Kostukovich E, Borisovsky I, Liu Z, Vinsavich A, Trush V, Quackenbush J (2003). TM4: a free, open-source system for microarray data management and analysis. Biotechniques.

[B4] White AM, Daly DS, Willse AR, Protic M, Chandler DP (2005). Automated Microarray Image Analysis Toolbox for MATLAB. Bioinformatics.

[B5] Tufte ER (1990). Envisioning Information.

[B6] The R-Project. http://www.r-project.org/.

[B7] Khan A JExcel API. http://www.andykhan.com/jexcelapi.

[B8] Mutton P Java EPS Graphics2D. http://www.jibble.org/epsgraphics.

[B9] Schuldiner M, Collins SR, Thompson NJ, Denic V, Bhamidipati A, Punna T, Ihmels J, Andrews B, Boone C, Greenblatt JF, Weissman JS, Krogan NJ (2005). Exploration of the function and organization of the yeast early secretory pathway through an epistatic miniarray profile. Cell.

